# Disambiguating Clinical Abbreviations by One-to-All Classification: Algorithm Development and Validation Study

**DOI:** 10.2196/56955

**Published:** 2024-10-01

**Authors:** Sheng-Feng Sung, Ya-Han Hu, Chong-Yan Chen

**Affiliations:** 1 Division of Neurology, Department of Internal Medicine, Ditmanson Medical Foundation Chia-Yi Christian Hospital, Chiayi City, Taiwan; 2 Department of Nursing, Fooyin University, Kaohsiung, Taiwan; 3 Department of Information Management, National Central University, 300 Zhongda Rd, Zhongli District, Taoyuan City, 32001, Taiwan, 886 34227151 ext 66560

**Keywords:** word sense disambiguation, electronic medical records, abbreviation expansion, text mining, natural language processing

## Abstract

**Background:**

Electronic medical records store extensive patient data and serve as a comprehensive repository, including textual medical records like surgical and imaging reports. Their utility in clinical decision support systems is substantial, but the widespread use of ambiguous and unstandardized abbreviations in clinical documents poses challenges for natural language processing in clinical decision support systems. Efficient abbreviation disambiguation methods are needed for effective information extraction.

**Objective:**

This study aims to enhance the one-to-all (OTA) framework for clinical abbreviation expansion, which uses a single model to predict multiple abbreviation meanings. The objective is to improve OTA by developing context-candidate pairs and optimizing word embeddings in Bidirectional Encoder Representations From Transformers (BERT), evaluating the model’s efficacy in expanding clinical abbreviations using real data.

**Methods:**

Three datasets were used: Medical Subject Headings Word Sense Disambiguation, University of Minnesota, and Chia-Yi Christian Hospital from Ditmanson Medical Foundation Chia-Yi Christian Hospital. Texts containing polysemous abbreviations were preprocessed and formatted for BERT. The study involved fine-tuning pretrained models, ClinicalBERT and BlueBERT, generating dataset pairs for training and testing based on Huang et al’s method.

**Results:**

BlueBERT achieved macro- and microaccuracies of 95.41% and 95.16%, respectively, on the Medical Subject Headings Word Sense Disambiguation dataset. It improved macroaccuracy by 0.54%‐1.53% compared to two baselines, long short-term memory and deepBioWSD with random embedding. On the University of Minnesota dataset, BlueBERT recorded macro- and microaccuracies of 98.40% and 98.22%, respectively. Against the baselines of Word2Vec + support vector machine and BioWordVec + support vector machine, BlueBERT demonstrated a macroaccuracy improvement of 2.61%‐4.13%.

**Conclusions:**

This research preliminarily validated the effectiveness of the OTA method for abbreviation disambiguation in medical texts, demonstrating the potential to enhance both clinical staff efficiency and research effectiveness.

## Introduction

The advent of electronic medical records (EMRs) has revolutionized data management in medical institutions by enabling the storage and collection of extensive patient data. EMRs integrate records and reports from various hospital departments, documenting diverse patient conditions and providing a comprehensive repository of information, including previous laboratory and examination reports, hospitalization and surgical procedure records, and medication histories [1-3]. EMRs contain two types of data: structured, such as physiological measurements, laboratory results, diagnostic and drug codes, and assessment scales, and unstructured, primarily consisting of textual medical records like surgical and imaging reports, pathology reports, and discharge summaries [4-10].

Recent studies have leveraged natural language processing (NLP) tools, including MetaMap, MedLEE, and Clinical Text Analysis and Knowledge Extraction System (cTAKES), to extract valuable patient information from EMRs’ clinical text [11-14]. These applications range from identifying specific medical concepts to complex analyses, such as discerning relationships between medical conditions or predicting patient outcomes and disease progression [10,15-19]. However, the prevalent use of abbreviations in clinical documents poses significant challenges for NLP in clinical decision support systems, as abbreviations often have multiple meanings depending on their context, and unstandardized or local abbreviations further complicate text interpretation [20,21]. This ambiguity impedes the extraction of meaningful information, affecting clinical decision support system performance and highlighting the need for effective methods for abbreviation disambiguation in clinical NLP applications.

Abbreviation disambiguation in NLP involves identifying the correct expansion of an abbreviation based on its context [22,23]. In this process, one-to-one (OTO) and one-to-all (OTA) approaches are two distinct strategies for resolving the meaning of abbreviations [24]. The OTO approach involves training a separate machine learning model for each specific abbreviation, learning its unique patterns and contextual cues to disambiguate its meaning. In contrast, the OTA approach uses a single machine learning model trained to disambiguate all abbreviations across various contexts.

The OTA approach in abbreviation disambiguation offers several advantages over the OTO approach. OTA is easier to scale, requiring the maintenance and updating of only a single model, whereas OTO necessitates multiple models for each abbreviation, making it less scalable. OTA is more efficient in terms of computational resources and ensures a uniform disambiguation approach, reducing inconsistencies. Additionally, OTA simplifies model management, streamlining changes and improvements. By learning general patterns and contextual cues applicable to various abbreviations, OTA enhances overall context understanding, making it suitable for applications with diverse abbreviation needs. This flexibility makes OTA particularly useful in the biomedical domain, where abbreviations can have varied meanings in different contexts.

This study aims to enhance the application of the OTA abbreviation disambiguation framework for clinical abbreviation expansion. We propose constructing an OTA disambiguation model by creating context-candidate pairs and refining word embeddings using Bidirectional Encoder Representations From Transformers (BERT) [25]. The model’s effectiveness was assessed based on its predictive performance on real clinical data for the task of clinical abbreviation expansion.

## Methods

### Data

This study conducted experimental evaluations using 3 datasets: 2 publicly available datasets and 1 independently collected from a regional hospital in Taiwan. The first dataset, the Medical Subject Headings Word Sense Disambiguation (MSH WSD) dataset, was extracted from MEDLINE abstracts [26]. The MSH WSD dataset comprises 203 polysemous words and is divided into three sections: abbreviation set, term set, and term/abbreviation set. The abbreviation set, containing 106 ambiguous acronyms, was selected as one of our investigated datasets. The second dataset, originating from the University of Minnesota (UMN), comprises deidentified clinical text sourced from the university’s hospitals [27]. The UMN dataset includes 440 frequently used abbreviations and acronyms, carefully selected from a pool of 352,267 dictated clinical notes. These two datasets are valuable resources for both NLP and medical informatics, particularly for disambiguation tasks within the health care domain [20,28,29].

Lastly, the Chia-Yi Christian Hospital (CYCH) dataset aggregates present illness data from patients at the Neurology Department of Ditmanson Medical Foundation Chia-Yi Christian Hospital. Abbreviation disambiguation results were validated by a neurologist. We narrowed the scope of abbreviations for evaluation and asked the doctor to mark the answers in advance. Specifically, we selected five frequently appearing abbreviations—ER, DM, CVA, PM, and PA—from both the UMN and CYCH datasets. We verified that the correct interpretations of abbreviations in the CYCH clinical documents matched the candidate sets for the UMN abbreviations. Due to manpower constraints, we limited our extraction to the first 1000 abbreviations for annotation by a physician. After removing one erroneous data entry and two initially overlooked abbreviations, we had a total of 998 sentences that included the five selected abbreviations. All 3 datasets were preprocessed and organized into the same format for subsequent model construction and evaluation. As shown in Table 1, the text “*...*He is status post a BK amputation on the right side and...” is partitioned into three parts: left, right, and target. Left denotes the text to the left of the target abbreviation, right represents the text to the right, and target indicates the target abbreviation. The remaining two fields include the correct expansion word for the target abbreviation (label) and the collection of all incorrect candidate expansion words (negs).

**Table 1.** Data schema after data preprocessing and an example sample text.

Field	Description	Example
Index	Document ID	1
Target	The target abbreviation	BK
Left	The text to the left of the target abbreviation	...He is status post a
Right	The text to the right of the target abbreviation	amputation on the right side and...
Label	The correct expansion word for the target abbreviation	below knee
Neg	The collection of all incorrect candidate expansion words. If there are multiple, separate them with commas.	BK(virus)

### Ethical Considerations

The study protocol received formal approval from the Ditmanson Medical Foundation Chia-Yi Christian Hospital Institutional Review Board (2022074). Patient identifiers were replaced by a unique study identification number to ensure confidentiality. Informed consent was thus exempted.

### The Proposed Framework

**Figure 1.**
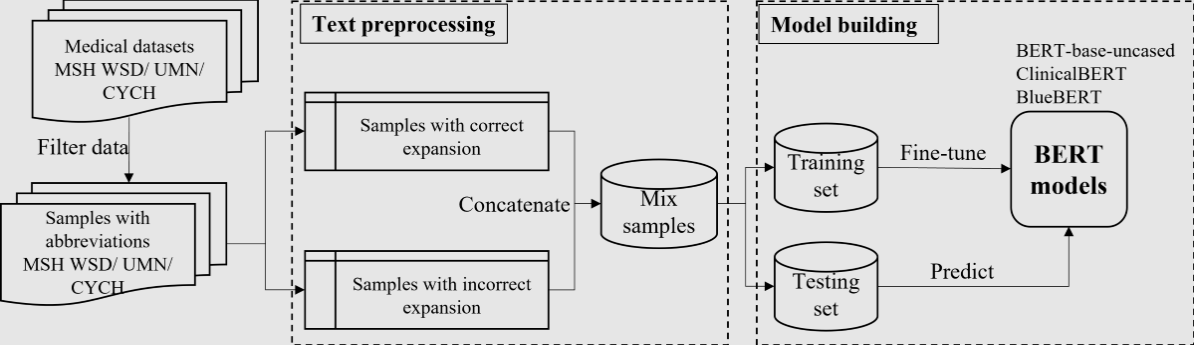
Research framework. BERT: Bidirectional Encoder Representations From Transformers; CYCH: Chia-Yi Christian Hospital; MSH WSD: Medical Subject Headings Word Sense Disambiguation; UMN: University of Minnesota.

Figure 1 illustrates the proposed framework. We begin by retrieving text containing polysemous abbreviations from the 3 investigated datasets. The polysemous abbreviations are kept in their original form and marked accordingly. Subsequent steps involve common text preprocessing techniques, such as converting text to lowercase and removing certain special symbols. The preprocessed text is then adjusted to meet the input format required by BERT. Finally, the processed text is divided into training and testing sets. Three existing pretrained BERT-based models, including BERT-base-uncased [25], ClinicalBERT [30], and BlueBERT [31], are chosen and fine-tuned using these datasets, and prediction results are subsequently generated for evaluation. BERT-base-uncased is specifically used due to the common inconsistencies in capitalization within clinical texts, where lowercase letters are frequently used, even at the beginning of sentences or in abbreviations.

### Text Preprocessing

This study converts the text into the context-candidate pair format and adjusts the word embedding values for BERT input. Specifically, we apply GlossBERT [32] to train our model using the samples consisting of abbreviations and all of their candidate expansions. If an abbreviation has *n* candidate expansions, with only one correct answer, we produce *n* samples. This includes one sample marked as the correct expansion (indicated as 1) and *n* – 1 samples marked as incorrect expansions (indicated as 0).

Before training, we use BERT’s tokenizer to convert text into WordPieces, breaking words such as “amputation” into [‘amp’, ‘##utation’]. Special tokens are then added: [CLS] at the start, [SEP] to separate sentences or differentiate sections, and [PAD] to equalize sequence lengths for batch processing. For instance, when processing the sentence “He is status post a BK amputation...” with “BK” having expansions “BK(virus)” and “below knee,” we generate two sequences: “[CLS] He is status post a BK amputation... [SEP] BK(virus) [SEP], 0” and “[CLS] He is status post a BK amputation... [SEP] below knee [SEP], 1.”

Due to BERT’s token limit of 512, sequences exceeding this are truncated. We manage sequence lengths by first converting text into WordPieces and adding necessary tokens. If the combined length of a sequence and its expansions exceeds BERT’s limit, we employ a first in, first out (FIFO) strategy to ensure compliance with the token restriction.

### BERT Tuning

Due to the limited dataset size, retraining a full BERT encoder was not feasible for this study. Instead, we fine-tuned existing pretrained models to assess our abbreviation disambiguation method. We selected two health care–related models, ClinicalBERT and BlueBERT, along with a generic BERT-base-uncased model as a baseline.

We adapted these models by adding a fully connected output layer. This layer consists of two linear layers and a rectified linear unit activation function, simplified as:


(1) $$y=f\left(\sum_{i=1}^{n}w_{k}x_{i}+b_{k}\right)$$

where $w_{k}$ represents the weights applied to inputs $x_{i}$, and $b_{k}$ is the bias term.

The output layer’s parameters are set as (50, 2), reflecting the size of the output from the previous layer and the number of classes (1 or 0). During prediction, the model calculates probabilities for each class. We focus primarily on the accuracy of the predictions for class 1, applying a softmax operation to enhance decision-making based on class 1’s probability scores. This process optimizes our approach to evaluating the effectiveness of the trained models in context-sensitive disambiguation tasks.

### Experimental Setup and Performance Measure

In our experimental evaluation, we aim to compare our proposed approach with several representative methods from prior studies on abbreviation disambiguation, focusing on model adaptability and performance across various datasets. The structure of our study is divided into two main parts.

Experiment 1 assesses the prediction performance of abbreviation expansion using both our proposed approach and baseline models (both OTO and OTA). We utilized two public datasets: MSH WSD and UMN. For MSH WSD, the OTO baselines included *k*-nearest neighbors [33], naive Bayes [26], and long short-term memory (LSTM) [34] models. For the UMN dataset, we referred to Wu et al [35] who used a combination of Word2Vec + support vector machine (SVM) as the OTO baseline. We further adapted this approach by substituting the original Word2Vec model with BioWordVec [36] (BioWordVec + SVM), which offers biomedical word embeddings via fastText, to better suit our study’s focus on clinical data. Each clinical note was represented as a 200-dimensional vector. For the OTA baseline models, we implemented non–sense-based methods using BERT/XLNet, as described by Kim et al [37], which include deepBioWSD_random embeddings_ and deepBioWSD_pretrained sense embeddings_. Additionally, we employed sense-based methods using bidirectional LSTM, outlined by Pesaranghader et al [38], specifically masked language modeling and permutation language modeling.

Experiment 2 evaluates the prediction performance of abbreviation disambiguation within the CYCH dataset, aiming to address abbreviation ambiguity in clinical contexts. This involved training models using the UMN dataset and testing them on the CYCH dataset. The experiment was designed to test how well the fine-tuning of pretrained models could adapt to a new hospital setting, using a combination of internal and external datasets to assess accuracy in a real-world clinical environment.

We conducted experiment 2 under two distinct scenarios to assess the adaptability and effectiveness of our model in handling abbreviation disambiguation. In the first scenario, we excluded CYCH text, utilizing only the UMN dataset for training. This approach tested the model’s ability to generalize from an external dataset to a new environment, applying it subsequently to 998 entries from the CYCH dataset. In the second scenario, we incorporated a small subset of CYCH text into the training process. This was designed to explore incremental learning, where the model adapts to new data while retaining previously learned information, thereby enhancing its predictive performance with minimal data and brief training periods.

Moreover, to maintain consistency and validity in our training process, it was crucial to ensure that all context-candidate pairs appeared in the training set. Consequently, the dataset was carefully screened before splitting, opting for a simple 9:1 ratio between the training and test sets instead of using cross-validation. To evaluate the model’s performance, we employed metrics such as accuracy, microaccuracy, and macroaccuracy. These metrics were derived from the confusion matrix for each abbreviation, providing detailed insights into the model’s efficacy across different contexts.

## Results

### Experiment 1

The results for experiment 1, using the MSH WSD dataset, are summarized in Table 2. For the OTO baselines, *k*-nearest neighbors achieved a macroaccuracy of 94.34%, with microaccuracy data unavailable. The naive Bayes method recorded a macroaccuracy of 93.86%, but microaccuracy was not reported. The LSTM method displayed both macro- and microaccuracy scores, which were 94.87% and 94.78%, respectively. For the OTA baselines, the sense-based deepBioWSD_random embeddings_ [38] achieved macro- and microaccuracy scores of 93.88% and 93.71%, respectively. The deepBioWSD_pretrained sense embeddings_ [38] improved prediction performance, with macro- and microaccuracy scores of 96.82% and 96.24%, respectively. Meanwhile, the non–sense-based methods, masked language modeling and permutation language modeling [37], recorded macroaccuracies of 95.89% and 96.83%, respectively, with microaccuracy not reported.

The BERT-base-uncased achieved macro- and microaccuracy scores of 93.64% and 93.38%, respectively. ClinicalBERT recorded a macroaccuracy of 94.77% and a microaccuracy of 94.59%. BlueBERT displayed a competitive performance with macro- and microaccuracies of 95.41% and 95.16%, respectively, on par with other evaluated methods. BlueBERT’s macroaccuracy was only slightly lower than the highest performing models, deepBioWSD_pretrained sense embeddings_ (96.82%) and permutation language modeling (96.83%), but higher than deepBioWSD_random embeddings_ (93.88%). This demonstrates BlueBERT’s robustness and effectiveness in sequence classification within this study.

The abbreviation disambiguation results for the UMN dataset, presented in Table 3, highlight the performance of various models. BlueBERT excelled, achieving macro- and microaccuracies of 98.4% and 98.22%, respectively, indicating its strong potential for disambiguation tasks. BERT-base-uncased and ClinicalBERT also showed strong performance, though slightly less than BlueBERT. In contrast, OTO-based models like Word2Vec + SVM and BioWordVec + SVM had lower accuracy scores, underscoring the advanced capabilities of the BERT models.

**Table 2.** Abbreviation disambiguation results (Medical Subject Headings Word Sense Disambiguation).

Method		Macroaccuracy (%)	Microaccuracy (%)
**One-to-one**			
	*k*-nearest neighbors [33]	94.34	—^a^
	Naive Bayes [26]	93.86	—
	Long short-term memory [34]	94.87	94.78
**One-to-all**			
	deepBioWSD_random embeddings_ [38]	93.88	93.71
	deepBioWSD_pretrained sense embeddings_ [38]	96.82	96.24
	Masked language modeling [37]	95.89	—
	Permutation language modeling [37]	96.83	—
	BERT-base-uncased	93.64	93.38
	ClinicalBERT	94.77	94.59
	BlueBERT	95.41	95.16

a Not applicable.

**Table 3.** Abbreviation disambiguation results (University of Minnesota).

Method (work)		Macroaccuracy (%)	Microaccuracy (%)
**One-to-one**			
	Word2Vec + SVM^a^ [35]	95.79	—^b^
	BioWordVec + SVM	94.27	—
**One-to-all**			
	Masked language modeling [37]	98.39	—
	Permutation language modeling [37]	98.28	—
	BERT-base-uncased	97.59	97.27
	ClinicalBERT	98.27	98.01
	BlueBERT	98.40	98.22

a SVM: support vector machine.

b Not applicable.

Overall, the proposed OTA method, especially when implemented using the pretrained BlueBERT model, outperformed the OTO-based approaches. The OTA method’s reliance on a single model, as opposed to the multiple models required by OTO methods, improves maintainability and scalability.

### Experiment 2

Table 4 displays the abbreviation disambiguation results for the CYCH dataset using BlueBERT. The table compares accuracy percentages for each abbreviation when trained exclusively on external data versus including incremental amounts of CYCH data (5 and 10 samples, respectively). For example, the model recorded a 62.07% accuracy for the abbreviation DM when trained without CYCH data. With the inclusion of CYCH data, the accuracy slightly improved to 70.27% with 5 samples but then slightly decreased to 100% with 10 samples. This trend of initial improvement followed by a marginal decline was observed for other abbreviations as well. Notably, the abbreviation PA showed a substantial increase in performance; it had 0% accuracy when trained without CYCH data but reached 100% accuracy when trained with either 5 or 10 CYCH samples.

**Table 4.** Abbreviation disambiguation results of the Chia-Yi Christian Hospital (CYCH) dataset.

Abbreviation	Training without CYCH data, accuracy (%)	Training with CYCH data	
		Accuracy, includes 5 documents (%)	Accuracy, includes 10 documents (%)
ER	97.91	98.03	97.75
DM	62.07	70.27	100
CVA	96.20	98.65	100
PM	77.27	100	100
PA	0.00	100	100

## Discussion

Automatic abbreviation disambiguation is crucial in clinical settings as it enhances the clarity and readability of medical records. By accurately interpreting abbreviations, it ensures that health care professionals have a precise understanding of patient information, facilitating accurate diagnoses and effective treatment plans. This automation also speeds up data processing, supports decision-making, and reduces errors, thereby improving overall health care delivery and patient safety.

Traditional OTO methods for abbreviation expansion involve constructing independent models for each abbreviation. Although this method offers high accuracy, it presents challenges in terms of maintenance and generalizability, complicating clinical applications due to the high number of models and associated maintenance costs. In contrast, this study proposes an approach that reduces the number of required models and offers better performance in clinical abbreviation restoration, thereby lowering both the operational and maintenance costs.

Compared to OTO, the OTA approach provides greater scalability, efficiency, and consistency, with a unified model that is easier to maintain and update. However, OTA approaches can be costly in terms of model retraining. Kim et al [37] highlighted that retraining the encoder necessitated high-end GPUs and substantial memory, requiring up to 14 days. Our study adopted a tuning approach using existing pretrained models, substantially cutting down training time to approximately an hour and a half by utilizing free online resources like the K80 GPU through Kaggle Notebook. This method effectively reduces both hardware and time costs, especially beneficial in clinical settings where frequent model updates may be necessary.

This study further demonstrates the practicality of this method in various hospital scenarios, particularly addressing cross-hospital and interdepartmental issues. Our incremental learning approach has been shown to significantly improve prediction results, thereby saving considerable retraining costs.

This study has the following limitations. First, although it preliminarily validates the exceptional effectiveness of the OTA method for abbreviation disambiguation in medical texts, the evaluation is limited by the size of the datasets used. More extensive and comprehensive clinical data are required before application to further validate this method. Second, our study is constrained by the maximum sequence length restriction of the BERT model. Longer clinical notes exceeding the 512-token limit must be truncated, risking the loss of information. Analysis shows that about 16.85% of the MSH WSD dataset and only 0.03% of the UMN dataset exceed this limit. The experimental results indicate superior accuracy for the UMN dataset; however, the performance for the MSH WSD dataset is lower, likely due to significant truncation of longer texts.

Additionally, in generating context-candidate pairs, we retain all candidates and use a FIFO approach for trimming the context. If an abbreviation appears at both the beginning and end of a context and exceeds the token limit, the FIFO method may remove the initial occurrence. Conversely, a last in, first out method could remove an abbreviation appearing at the end. If the same abbreviation carries different meanings in different parts of the text, identical context-candidate pairs may be created post trimming, potentially distorting model training and leading to incorrect predictions.

This study presents an innovative approach to the disambiguation and expansion of abbreviations in clinical medical texts by utilizing context-candidate pairs and the BERT model. This method enhances the readability of medical texts, improving the efficiency of clinical staff who review EMRs and saving time for cross-disciplinary researchers analyzing clinical data, thereby increasing the effectiveness of their studies. Given that clinical medical texts are replete with abbreviations, accurate disambiguation is essential for improving text clarity and usability. Automating this process greatly assists both medical professionals and researchers. The successful application of this model on the investigated datasets underscores its effectiveness and establishes it as a valuable reference for future research in clinical abbreviation expansion.

## Abbreviations

BERT: Bidirectional Encoder Representations From Transformers
cTAKES: Clinical Text Analysis and Knowledge Extraction System
CYCH: Chia-Yi Christian Hospital
EMR: electronic medical record
FIFO: first in, first out
LSTM: long short-term memory
MSH WSD: Medical Subject Headings Word Sense Disambiguation
NLP: natural language processing
OTA: one-to-all
OTO: one-to-one
SVM: support vector machine
UMN: University of Minnesota
